# High-Precision Monitoring of Average Molecular Weight of Polyethylene Wax from Waste High-Density Polyethylene

**DOI:** 10.3390/polym12010188

**Published:** 2020-01-10

**Authors:** Zhouchao Guo, Xia Lan, Ping Xue

**Affiliations:** School of Mechanical and Electrical Engineering, Beijing University of Chemical Technology, Beijing 100029, China; gzctaopu@yeah.net (Z.G.); lanxia@mail.buct.edu.cn (X.L.)

**Keywords:** high-precision monitoring, polyethylene wax, HDPE pyrolysis, extrusion

## Abstract

High-density polyethylene (HDPE) is a major component of polyethylene waste, yet only under 29.9% of waste HDPE is recycled. As an important additive, polyethylene wax (PEW) is increasingly used in many industries such as plastics, dyes, and paints. The preparation of PEW has received considerable interest because recycling and precisely controllable production can bring huge economic benefits. In this study, to recycle waste HDPE, a single screw extruder was innovatively combined with a connecting pipe to prepare PEW from the pyrolysis of waste HDPE. Using a test platform, PEWs were prepared under different pyrolysis temperatures and screw speeds, and corresponding number-average molecular weights (NAMWs) of PEWs were measured. To precisely monitor NAMW of PEW, a program was developed in MATLAB. First, the relationship between NAMW and pyrolysis ratio was obtained, and a measure-point-independence verification was conducted. Then, modified Arrhenius equations and time-dependent pyrolysis temperature were for the first time introduced into the HDPE pyrolysis model. Furthermore, the screw-speed-dependent inverse method was proposed and validated for high-precision monitoring of NAMW of PEW from the pyrolysis of waste HDPE by extrusion. PEW of desired molecular weight was able to be precisely obtained from waste HDPE.

## 1. Introduction

Polyethylene (PE) materials have become more and more important to everyday life, being used in a wide scope of applications from pipe to packaging to fiber. However, with their increasing use, PE waste makes up a large proportion of all municipal solid waste, yet only a small proportion of PE waste is recycled. High-density polyethylene (HDPE) is a major component of PE waste, yet only under 29.9% of waste HDPE is recycled [1]. Thus, developing effective reusing techniques for HDPE waste is important.

Although the volume of waste HDPE materials can be mitigated by waste minimization [2] and mechanical recycling [3], large-scale solutions are required for the large magnitude of waste HDPE materials [4]. Among the solutions available for a large-scale recycling of waste HDPE, are stand out pyrolysis and gasification [5,6]. Gasification technologies have a long history of research [7], and the gasification of heterogeneous mixtures of waste PE has been realized [8]. However, compared to gasification, pyrolysis requires lower energy and produces lower amounts of pollutants because it operates at lower temperature and generates less CO_2_ [9]. In addition, the pyrolysis technology can be used to produce functional waxes or monomer materials with appropriate reaction equipment and operating conditions [10]. Using a system of fixed bed-fixed bed reactors, Akubo and coworkers obtained aromatic fuel oils from HDPE pyrolysis [11]. As an important additive, polyethylene wax (PEW) is increasingly used in many industries such as plastics, dyes, and paints. PEW of appropriate molecular weight can be used as a release agent in plastic processing.

Pyrolysis, heating the material under non-oxidative condition, is an attractive waste recycling method because of its simplicity and ability to reprocess heterogeneous waste [12,13]. However, inhomogeneous heating can occur in existing equipment of PE pyrolysis, such as kettle type [14] or tube type [15]. The properties of pyrolysis products can be affected for the reason that inhomogeneous heating can induce hot spots, excessive pyrolysis, and even carbon deposition of PEW. In such cases, single screw extruder (SSE) is considered as a new and effective method to continuously prepare PEW with comparatively homogeneous heating [16]. However, a greater understanding of the mechanism of HDPE pyrolysis and a precise monitoring of molecular weight of PEW are needed to continue the development of this important technology.

The high molecular weight and polydispersity of HDPE cause its pyrolysis reaction network to often involve hundreds of species and reactions. The complex reaction network often gives rise to a broad product spectrum [17]. This complexity of the pyrolysis reactions makes it difficult to use experimental methods to obtain detailed mechanistic insight. In this situation, creating a mathematical model is a useful tool for obtaining insight into the complex mechanism of HDPE pyrolysis. PE pyrolysis has been studied for a long history [18,19,20]; however, unresolved questions surrounding the mechanism and kinetics still exist, most likely resulting from the difficulty in analyzing the diverse product spectrum [21]. The competition between inter- and intra-molecular hydrogen transfer reactions has attracted a variety of studies [22,23,24,25]; however, their conclusions about the relative importance of the two reaction modes are inconsistent.

In general, there are three reaction pathways for polymer pyrolysis: (1) Unzipping, which yields monomer from the polymer; (2) backbiting (BB), which yields a series of low molecular weight products; and (3) random scission (RS), which yields a diverse array of low molecular weight products [26]. The biggest difference between BB and RS pathways is just the competition between inter- and intra-molecular hydrogen transfer reactions. Since an equally stable secondary carbon radical generates for every mid-chain hydrogen, PE is especially susceptible to both BB and RS pathways. Faravelli and coworkers developed a series of versions of a mechanistic model for PE pyrolysis [27,28,29]. Their initial model, focusing on the total product yield, only included the RS pathway [27]. They improved this model with the addition of the BB pathway so that detailed product distributions were able to be modeled [28]. With the competition between RS and BB analyzed in their most recent modeling work, they found that RS was more important in PE pyrolysis [29]. Levine and Broadbelt developed a detailed mechanistic model for HDPE pyrolysis, allowing the tracking of specific products during the pyrolysis procedure of HDPE [30]. Likewise, continuous improvements have been done in the treatment of landfill waste plastics by means of an artificial neural network [31] and a multiple linear regression model [32]. However, the modified Arrhenius equation and time-dependent pyrolysis temperature are rarely reported in the literature. The method of monitoring number-average molecular weight (NAMW) of PEW from available observations of pyrolysis temperature and screw speed is new. In addition, an inverse method is rarely used to estimate NAMW of PEW, and high-precision monitoring of NAMW of PEW from the pyrolysis of waste HDPE by extrusion is new.

In this study, to recycle waste HDPE, a single screw extruder was innovatively combined with a connecting pipe to prepare PEW from the pyrolysis of waste HDPE. Using a test platform, PEWs were prepared under different pyrolysis temperatures and screw speeds, and corresponding NAMWs of PEWs were measured. To monitor NAMW of PEW, a program was developed in MATLAB. First, the relationship between NAMW and pyrolysis ratio was obtained based on RS reaction pathway, and measure-point-independence verification was added into the program. Then, the modified Arrhenius equation and time-dependent pyrolysis temperature were for the first time introduced into the HDPE pyrolysis model for a higher monitoring precision. Furthermore, the inverse method with screw-speed-dependent reaction rate constant was proposed and validated for high-precision monitoring of NAMW of PEW from the pyrolysis of waste HDPE by extrusion.

## 2. Test Procedure

### 2.1. Test Platform

**Figure 1:**
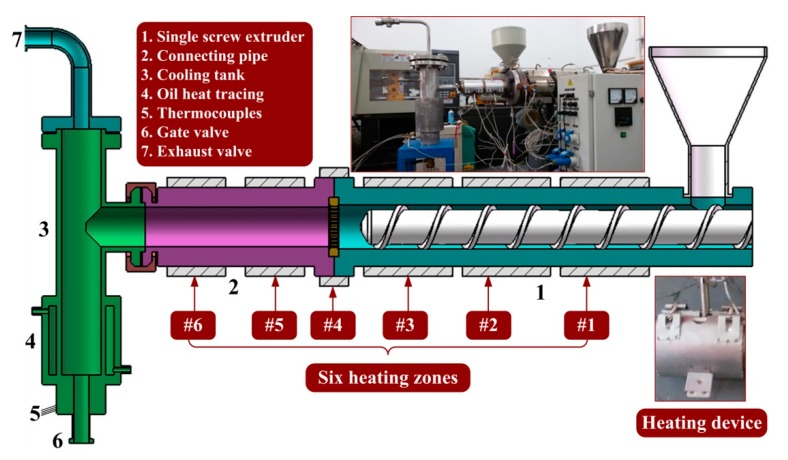
Flowchart of test platform.

Using a test platform, PEW was prepared from the pyrolysis of waste HDPE in a single screw extruder (SSE) and a connecting pipe. A flowchart of the test platform is shown in Figure 1. It can be seen that waste HDPE from a feed hopper flows through a SSE and a connecting pipe, and then flows into a cooling tank. There are four heating zones (Zone #1, #2, #3, and #4) for the SSE and two heating zones (Zone #5 and #6) for the connecting pipe. HDPE is pyrolyzed in the SSE and the connecting pipe, while PEW is cooled and stored in the cooling tank.

To guarantee a continuous pyrolysis of HDPE in the SSE, the pressure in the cooling tank should not be too high. Generated gas during the pyrolysis of HDPE flows from a top discharge of the cooling tank, passes through an exhaust valve, and finally flows into a device for tail gas treatment. The exhaust valve should be a check valve in case air flows into the cooling tank and PEW of high temperature oxidizes or even spontaneously combusts. In addition, there is a metallic pressure sealing ring between the connecting pipe and the cooling tank, which also guarantees the inert condition (i.e., absence of oxygen). On the outer surface of the cooling tank, we use a sleeve in which conduction oil with high temperature flows to avoid the clot of liquid PEW. In addition, thermocouples and data acquisition unit are used to measure the temperature of PEW at the bottom outlet of the cooling tank.

### 2.2. Preparation of Polyethylene Wax

**Figure 2:**
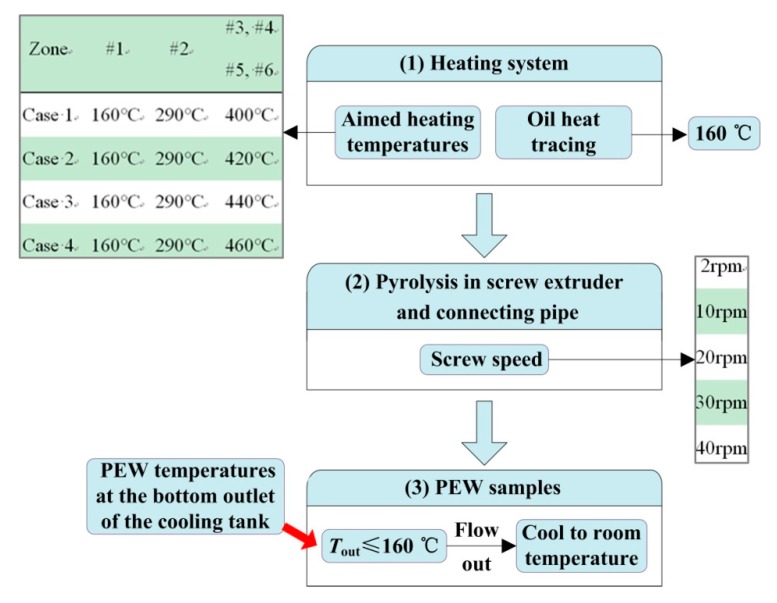
Flowchart of preparation of polyethylene wax (PEW).

HDPE (ME9180), of which the number-average molecular weight is 13,295, is provided by LG Co. (Seoul, Korea). The heating temperatures of the SSE and the connecting pipe under four cases are shown in Figure 2, while the temperature of the oil heat tracing is set as 160 °C. The screw speed of the SSE is set as 2, 10, 20, 30, and 40 rpm, respectively. The gate valve (labeled 6 in Figure 1) will be open when the temperature of liquid PEW at the bottom outlet of the cooling tank is not higher than 160 °C. Liquid PEW flows out, and then it is cooled to room temperature to prepare the PEW samples.

Under different screw speeds, the flow time of material from the inlet of the SSE to the outlet of the connecting pipe was measured and the flow time of material from the inlet of the SSE to the inlet of Heating Zone #3 was calculated. Then, the pyrolysis time of material was obtained by calculating the difference between the two flow times. Table 1 shows the correspondence between pyrolysis time and screw speeds.

### 2.3. Characterization of Polyethylene Wax

**Figure 3:**
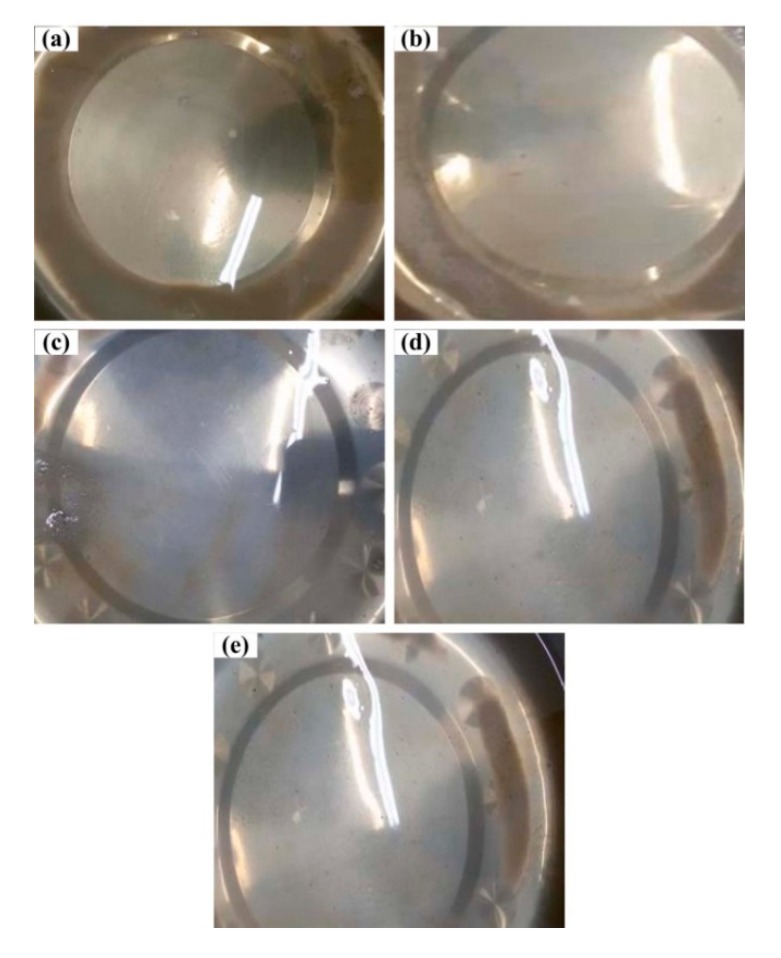
Liquid PEWs prepared under different screw speeds at the pyrolysis temperature of 693 K. (**a**) *n* = 2 rpm, (**b**) *n* = 10 rpm, (**c**) *n* = 20 rpm, (**d**) *n* = 30 rpm, (**e**) *n* = 40 rpm.

Liquid PEWs flow out when their temperatures are not higher than 160 °C, the colors of which are shown in Figure 3 under different screw speeds at the pyrolysis temperature of 693 K. With increasing the screw speed, it can be seen that the color of PEW lightens gradually. At a lower screw speed, more molecular chains of low molecular weight generate, and the PEW of lower molecular weight is more likely to be oxidized to yield a darker color.

**Figure 4:**
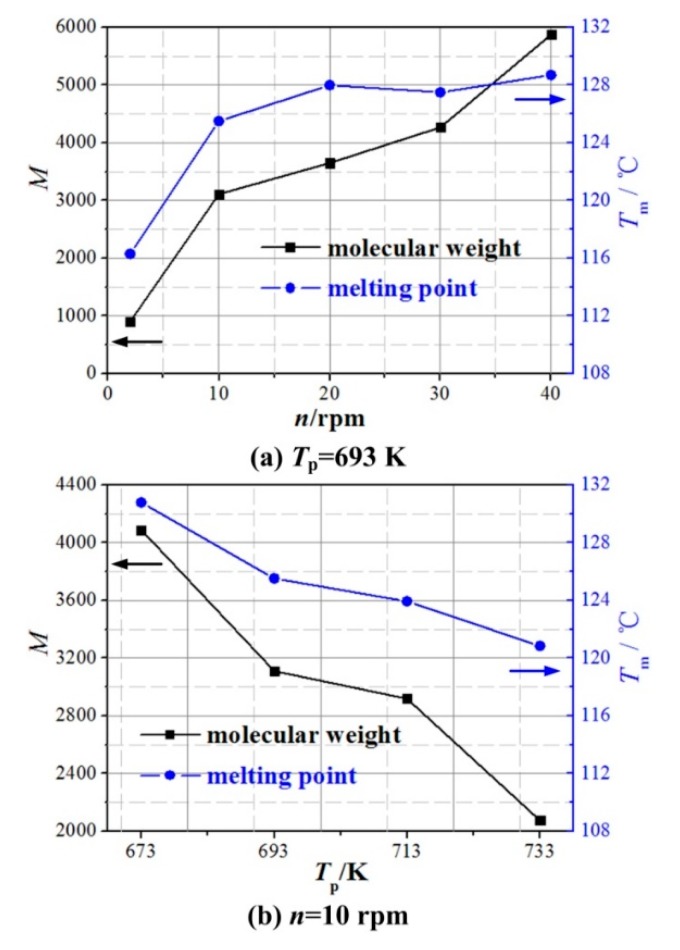
Effects of screw speed and pyrolysis temperature on the number-average molecular weight (NAMW) and melting point of PEW. (**a**) screw speed, (**b**) pyrolysis temperature.

DSC (Q600, Thermal Analysis Co., Newtown, PA, USA) and GPC (PL-GPC 220, Polymer Laboratories Co., Salop, UK) were used to measure the melting points, molecular weights, and molecular weight distributions of aforementioned PEWs. In addition, a universal calibration was used in this study. As shown in Figure 4, the effects of screw speed and pyrolysis temperature on the number-average molecular weight (NAMW) and melting point of PEW can be summarized as (1) the molecular weight and melting point of PEW increase with increasing the screw speed and decrease with increasing the pyrolysis temperature and (2) low screw speed and high pyrolysis temperature are expected to obtain the PEW of low molecular weight.

**Figure 5:**
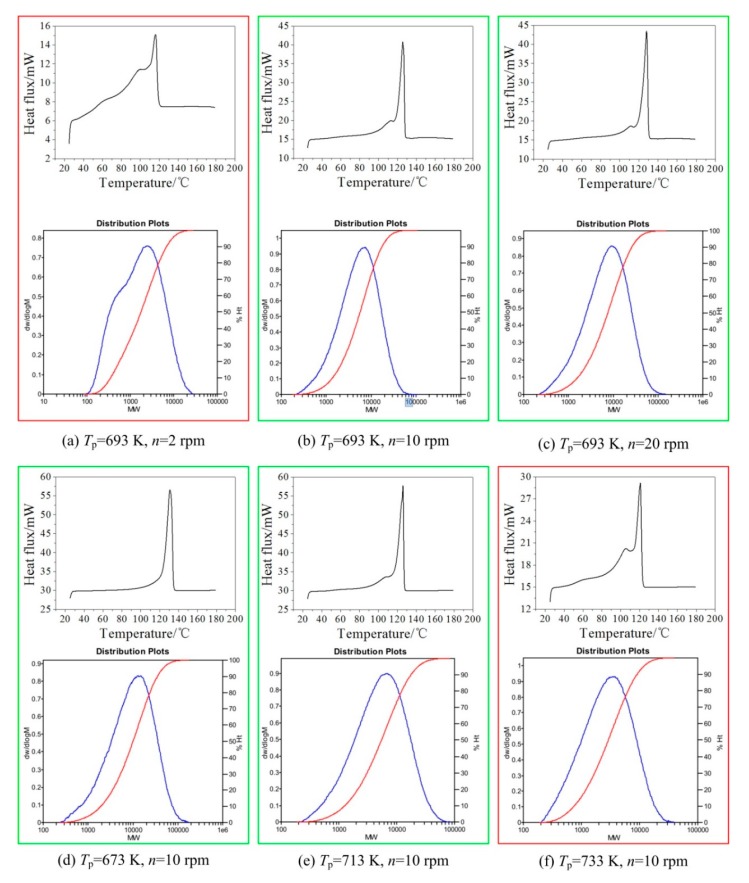
Differential scanning calorimetry (DSC) curves and molecular weight distribution plots of PEWs under different screw speeds and pyrolysis temperatures. (**a**) *T*_p_ = 693 K, *n* = 2 rpm, (**b**) *T*_p_ = 693 K, *n* = 10 rpm, (**c**) *T*_p_ = 693 K, *n* = 20 rpm, (**d**) *T*_p_ = 673 K, *n* = 10 rpm, (**e**) *T*_p_ = 713 K, *n* = 10 rpm, (**f**) *T*_p_ = 733 K, *n* = 10 rpm.

However, a too low screw speed or too high pyrolysis temperature is not suggested not only because of low efficiency or high energy consumption but also because of a too wide melting peak and many PEWs of low molecular weights as shown in red boxes in Figure 5. In engineering practice, a too wide melting peak and many PEWs of low molecular weights can result in poor thermal properties. It can be seen from Figure 5a–c, that for a fixed pyrolysis temperature, a wide melting peak (even double melting peaks) and many PEWs of low molecular weights occur when the screw speed reduces to 2 rpm, i.e., the screw speed of 2 rpm is not desired. Likewise, for a fixed screw speed in Figure 5d–f, the wide melting peak (even double melting peaks) and many PEWs of low molecular weights in Figure 5f indicate that the pyrolysis temperature of 733 K is not desired.

To sum up, both the screw speed and the pyrolysis temperature have an important effect on the NAMW of PEW. PEW of a desired NAMW and a narrow melting peak can be prepared from the pyrolysis of waste HDPE under appropriate screw speed and pyrolysis temperature. It can be helpful to develop the method of precisely monitoring NAMW of PEW from the available observations of pyrolysis temperature and screw speed.

## 3. Theoretical Analysis of Pyrolysis Procedure

### 3.1. Pyrolysis Model

**Figure 6:**
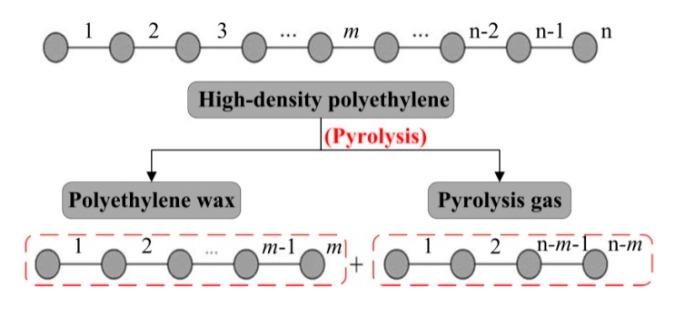
Pyrolysis scheme of random scission for high-density polyethylene (HDPE).

Figure 6 shows the pyrolysis scheme of random scission for HDPE. To simplify our model, it is assumed that: (1) the pyrolysis occurs at any node of the molecular chain with an equal probability because of the quasi-linear molecular structure of HDPE; (2) based on the principle of random process, the pyrolysis can be expressed with the same reaction dynamic equation using the same reaction order; (3) the pyrolysis time does not vary with the changes in viscosity stemming from the different pyrolysis temperatures and only depends on the screw speed; and (4) the reaction rate constant is uniform in the whole heating zones #3 to #6 of the SSE and the connecting pipe.

### 3.2. Theoretical Calculation of Average Carbon-Atom Number of PEW

The governing equation for the pyrolysis of HDPE is:

(1) $$\frac{dr}{dt}=(1-r{)}^{v}\cdot k(T_{p})$$

It is assumed that the total number of molecular chain nodes that are not pyrolyzed is *b*. At time *t* = 0 s, there are no pyrolyzed nodes, and the pyrolysis ratio *r* = 0. Therefore, the pyrolysis ratio *r* can be defined by:

(2) $$r=1-\frac{b(r)}{b(0)}$$

where *b*(0) and *b*(*r*) represent the numbers of not pyrolyzed nodes at pyrolysis ratio of 0 and *r*. The pyrolysis ratio of HDPE is *r* at time *t*. At pyrolysis ratio *r*, it is assumed that the total number of molecule chains with *m* carbon atoms is *N_m_*. Therefore, we obtain the following equation:

(3) $$b(r)=\sum_{m=1}^{n}\left(\left(m-1)\cdot N_{m}(r\right)\right)$$

When the pyrolysis ratio increases from *r* to r+Δr, the increased number of molecule chains with *m* carbon atoms is:

(4) $$\Delta N_{m}^{+}=b(0)\cdot \Delta r\cdot \sum_{i=m+1}^{n}\left(N_{i}(r\right)\cdot \frac{i-1}{b(r)}\cdot \frac{2}{i-1})=\frac{2\Delta r}{1-r}\sum_{i=m+1}^{n}N_{i}(r)$$

and the decreased number of molecule chains with *m* carbon atoms is:

(5) $$\Delta N_{m}^{-}=b(0)\cdot \Delta r\cdot \frac{\left(m-1)N_{m}(r\right)}{b(r)}=(m-1)N_{m}(r)\frac{\Delta r}{1-r}$$

Therefore, at pyrolysis ratio r+Δr, the total number of molecule chains with *m* carbon atoms is:

(6) $$N_{m}(r+\Delta r)=N_{m}(r)+\frac{2\Delta r}{1-r}\sum_{i=m+1}^{n}N_{i}(r)-(m-1)N_{m}(r)\frac{\Delta r}{1-r}$$

and Equation (6) can be rewritten as an ordinary differential Equation:

(7) $$\frac{dN_{m}(r)}{dr}=\frac{2}{1-r}\sum_{i=m+1}^{n}N_{i}(r)-\frac{\left(m-1)N_{m}(r\right)}{1-r}$$

By solving Equation (7), the following equation is obtained:

(8) $$N_{m}(r)=C\cdot r^{2}\cdot {\left(1-r\right)}^{m-1}$$

where *C* is a constant number.

In this study, the evaporation temperature equals the temperature of the oil heat tracing, i.e., 433 K. According to Wallis and Bhatia [33], the relationship between the maximum carbon-atom number of pyrolysis gas *m_v_* and the evaporation temperature *T*_v_ is:

(9) $$m_{v}=9\times 10^{-5}T_{v}{}^{2}-0.0317T_{v}+5.1497$$

At pyrolysis ratio *r*, the average carbon-atom number of PEW $\bar{M}(r)$ can be given by:

(10) $$\bar{M}(r)=\frac{\sum_{m=m_{v}}^{n}mN_{m}(r)}{\sum_{m=m_{v}}^{n}N_{m}(r)}=\frac{\sum_{m=m_{v}}^{n}m{\left(1-r\right)}^{m-1}}{\sum_{m=m_{v}}^{n}{\left(1-r\right)}^{m-1}}$$

where $\sum_{m=m_{v}}^{n}m{\left(1-r\right)}^{m-1}$ and $\sum_{m=m_{v}}^{n}{\left(1-r\right)}^{m-1}$ can be obtained by Equations (11) and (12):

(11) $$\begin{array}{ll} \sum_{m=m_{v}}^{n}m{\left(1-r\right)}^{m-1} & ={\left(1-r\right)}^{m_{v}-1}\left\{m_{v}\sum_{j=1}^{n-m_{v}+1}{\left(1-r\right)}^{j-1}+(1-r)\sum_{j=1}^{n-m_{v}}j{\left(1-r\right)}^{j-1}\right\}\\ & \approx {\left(1-r\right)}^{m_{v}-1}\left(\frac{m_{v}}{r}+\frac{1-r}{r^{2}}\right)\left(n\to +\infty \right) \end{array}$$

(12) $$\sum_{m=m_{v}}^{n}{\left(1-r\right)}^{m-1}=\frac{{\left(1-r\right)}^{m_{v}-1}}{r}\left[1-(1-r{)}^{n-m_{v}+1}\right]\approx \frac{{\left(1-r\right)}^{m_{v}-1}}{r}\left(n\to +\infty \right)$$

Therefore, Equation (10) can be rewritten as:

(13) $$\bar{M}(r)\approx m_{v}+\frac{1}{r}-1$$

## 4. Monitoring Procedure for Average Molecular Weight of Polyethylene Wax

**Figure 7:**
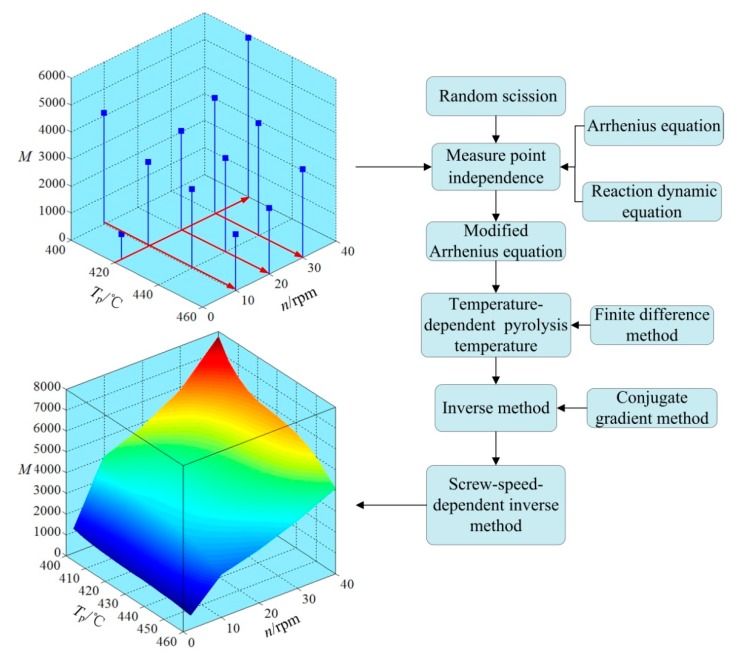
Procedure to monitor NAMW of PEW from the pyrolysis of waste HDPE by extrusion.

The procedure to precisely monitor NAMW of PEW from the pyrolysis of waste HDPE by extrusion is summarized as follows and shown in Figure 7:

Step 1. Measure point independence verification is conducted based on the pyrolysis mechanism of random scission, Arrhenius equation, and the reaction dynamic equation by using experimental data at measure points N1, N2, N3, N6, N8, N9, N10, and N12 (refer to Table 2).

Step 2. The modified Arrhenius equation is used to fit the relationship between the reaction rate constant $k(T_{p})$ and the pyrolysis temperature $T_{p}$.

Step 3. The effect of the inevitable small fluctuations of pyrolysis temperature stemming from a temperature control system on monitoring precision is studied.

Step 4. With the results of the modified Arrhenius equation (*o* = 3) used as initial values, an inverse estimation based on the conjugate gradient method is introduced for higher precision.

Step 5. A screw-speed-dependent inverse method is proposed to further improve the monitoring precision of NAMW of PEW for Ω = [2, 40] × [400, 460].

### 4.1. Arrhenius Equation

According to Westerhout R W J [34], a first-order reaction dynamic equation can be used because the conversion ratio of the feedstock (waste HDPE) is higher than 98% during our tests. Therefore, in this study, Equation (1) can be rewritten as:

(14) $$\frac{dr}{dt}=(1-r)\cdot k(T_{p})$$

The initial condition is:

(15) r(0)=0

By solving Equations (14) and (15), we obtain:

(16) $$r=1-e^{-k(T_{p})\cdot t}$$

The pyrolysis time *t* in Equation (16) can be obtained from the screw speed *n* (refer to Table 1), while the pyrolysis ratio *r* in Equation (16) can be obtained from the evaporation temperature *T*_v_ and the average carbon-atom number of PEW $\bar{M}(r)$ using Equations (9) and (13). Thus, the reaction rate constant $k(T_{p})$ can be easily obtained. After taking logarithm of Arrhenius equation, we obtain:

(17) $$\ln k(T_{p})=\ln A_{1}-\frac{E}{RT_{p}}$$

Based on a linear regression, the factor before exponent *A*_1_ and the activation energy *E* can be determined using our partial test data.

### 4.2. Measure-Point-Independence Verification

Table 2 shows the arrangements of measure points under different values of *p*, where *p* represents the number of measure points. It is worth mentioning that a measure point here represents one group of test: pyrolysis temperature, screw speed, and corresponding NAMW of PEW; therefore, there are twelve measure points for our tests in total.

**Figure 8:**
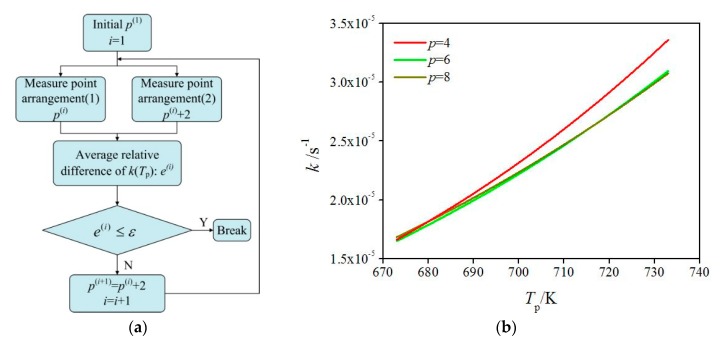
Measure-point-independence verification. (**a**) flowchart of measure-point-independence verification, (**b**) comparisons of Arrhenius equations obtained at four, six, and eight measure points.

To get a measure-point-independent solution, we added measure-point-independence verification into our program. Figure 8a shows a flowchart of the measure-point-independence verification. During the measure-point-independence verification, based on a linear regression, we solved the Arrhenius equation for the pyrolysis of HDPE under a series of measure point arrangements. Figure 8b shows the Arrhenius equations obtained under different measure point arrangements. It can be seen that the results at *p* = 4, 6, and 8 show the same trend. Careful comparisons of the results indicate that the average relative difference is smaller than 4.38% between *p* = 4 and *p* = 6 and smaller than 0.82% between *p* = 6 and *p* = 8.

### 4.3. Modified Arrhenius Equation

**Figure 9:**
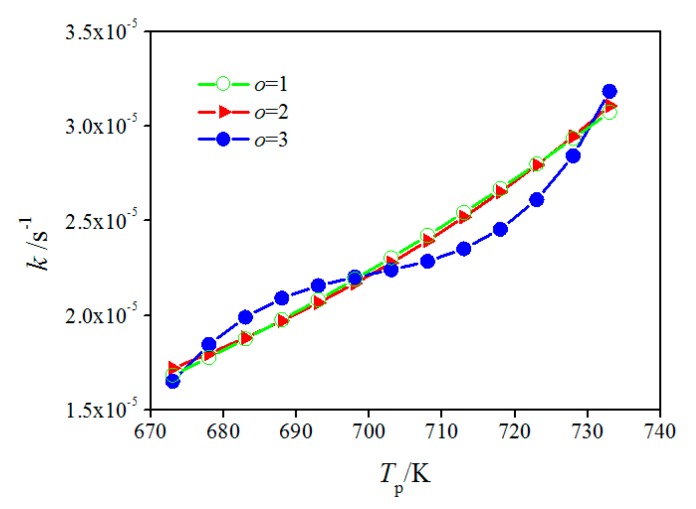
Comparisons of initial and modified Arrhenius equations.

During the monitoring procedure, the measured NAMWs of PEWs at measure points N1, N2, N3, N6, N8, N9, N10, and N12 (refer to Table 2) were used as input data. To validate the monitoring results, we compared the measured and calculated NAMWs of PEWs at measure points N4, N5, N7, and N11 (refer to Table 2). As shown in Figure 9, the reaction rate constants for *o* = 1, 2, and 3 show the same trend; however, the curves for *o* = 1 and 2 are similar, while the curve for *o* = 3 is obviously different from them. The curve for *o* = 3 is smoother than those for *o* = 1 and 2 for the reason that the modified Arrhenius equation with a higher-order allows more degrees of freedom. Thus, the curve for *o* = 3 is expected to be more approaching to the real relationship between reaction rate constant and pyrolysis temperature than those for *o* = 1 and 2. The relative errors of NAMW of PEW at four measure points (N4, N5, N7, and N11) for initial and modified Arrhenius equations are shown in Table 3. It can be seen that the mean relative error of NAMW of PEW at aforementioned measure points reduces to 3.6% from 8.1%.

### 4.4. Time-Dependent Pyrolysis Temperature

During our tests, the small fluctuations of pyrolysis temperature stemming from the temperature control system were inevitable. To study the effect of the time-dependent pyrolysis temperature on monitoring precision, the fluctuations of pyrolysis temperature were assumed as the following Equation:

(18) $$\Delta T_{p}=A_{2}\sin\left(2\pi /l\cdot t\right)$$

**Figure 10:**
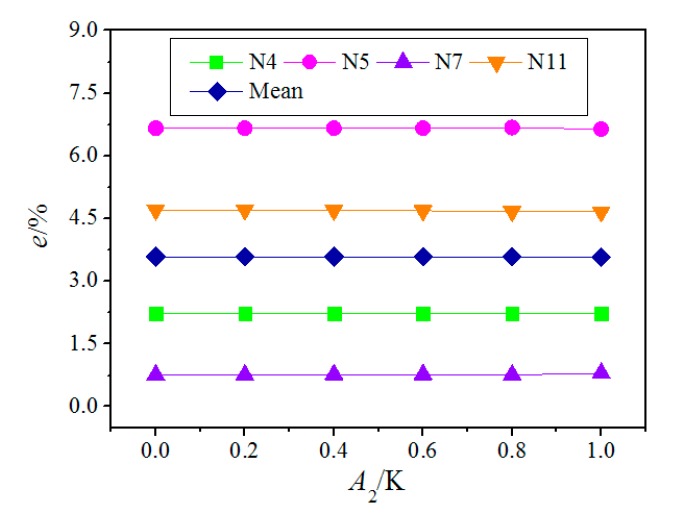
Effect of fluctuation amplitude of pyrolysis temperature on monitoring precision. *A*_2_: (the fluctuation amplitude of pyrolysis temperature); *e*: (the relative errors of NAMW of PEW).

When the pyrolysis temperature varied as time, the reaction rate constant $k(T_{p})$ in Equation (14) should not be regarded as a time-independent constant, i.e., Equation (16) was no longer valid. In this situation, an analytical solution was no longer easy; therefore, we solved the Equation (14) by the finite difference method. The NAMWs of PEWs were estimated at *l* = 60 s under different amplitudes of temperature fluctuation as shown in Figure 10. It can be seen that the relative errors of NAMW of PEW are similar when the amplitude of temperature fluctuation is not larger than 1 K, an indication that the time-dependent pyrolysis temperature will not improve the precision much but increase the computational cost. Larger fluctuations were not considered for the reason that the controlment precision of the pyrolysis temperature was within 1 K.

### 4.5. Inverse Method

During an inverse estimation, the reaction rate constant $k(T_{p})$ is assumed, and the corresponding NAMWs of PEWs are calculated. The calculated NAMWs of PEWs are compared with the measured values. Because the inverse estimation can be treated as an optimization problem, we use an objective function based on the least-squares method for the inverse estimation:

(19) $$J=\sum_{i=1}^{Z}{\left[M_{i,\mathrm{cal}}-M_{i,\mathrm{mea}}\right]}^{2}$$

J is a function of $M_{i,\mathrm{cal}}$, and $M_{i,\mathrm{cal}}$ varies with $k(T_{p})$. Therefore, J is a function of $k(T_{p})$, and we obtain the optimization problem:

(20) $$\min J=J\left[k(T_{p})\right]$$

The optimization problem can be solved by the following iterative format:

(21) $$k{\left(T_{p}\right)}^{\left(j+1\right)}=k{\left(T_{p}\right)}^{\left(j\right)}+\beta^{\left(j\right)}d^{\left(j\right)}$$

When we use the conjugate gradient method, $d^{\left(j\right)}$ is given by the following equation:

(22) $$d^{\left(j\right)}=-\nabla J(k{\left(T_{p}\right)}^{\left(j\right)})+\frac{{\left\|\nabla J(k{\left(T_{p}\right)}^{\left(j\right)})\right\|}^{2}}{{\left\|\nabla J(k{\left(T_{p}\right)}^{\left(j-1\right)})\right\|}^{2}}d^{\left(j-1\right)}$$

and $\beta^{\left(j\right)}$ should meet the following condition:

(23) $$J(k{\left(T_{p}\right)}^{\left(j\right)}+\beta^{\left(j\right)}d^{\left(j\right)})=\underset{\beta \geq 0}{\min}J(k{\left(T_{p}\right)}^{\left(j\right)}+\beta d^{\left(j\right)})$$

It is clear that $k{\left(T_{p}\right)}^{\left(1\right)}$ is not the true value; however, with the iteration process, $k{\left(T_{p}\right)}^{\left(j\right)}$ can approach the true value when the stopping criterion is met:

(24) $$\left\|d^{\left(j\right)}\right\|\leq \varepsilon$$

For the aforementioned inverse estimation, the reaction rate constant $k(T_{p})$ is only a function of the pyrolysis temperature $T_{p}$. However, the pyrolysis reaction is under both external heater and internal shear heat, and the screw speed should have an effect on the reaction rate constant; i.e., it is more reasonable that the reaction rate constant $k(T_{p},n)$ is a function of the pyrolysis temperature $T_{p}$ and the screw speed *n*. Therefore, $k(T_{p})$ in Equations (18)–(23) should be replaced with $k(T_{p},n)$.

**Figure 11:**
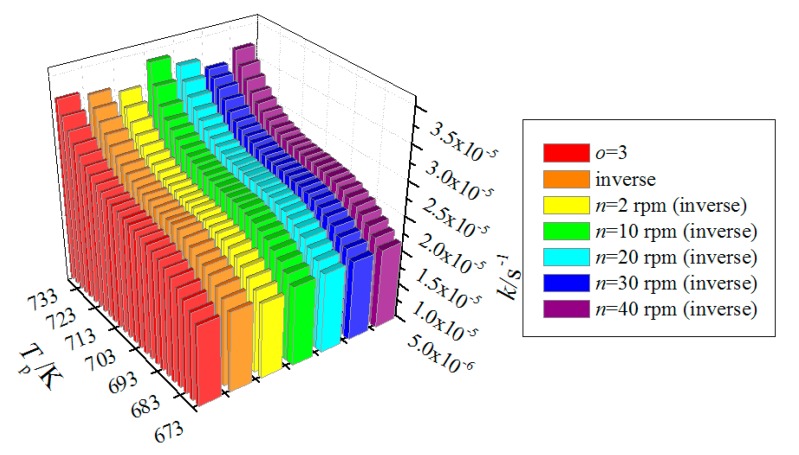
Comparisions of reaction rate constants between the modified Arrhenius equation (*o* = 3), inverse method, and screw-speed-dependent inverse method.

Reaction rate constants for the modified Arrhenius equation (*o* = 3), inverse method, and screw-speed-dependent inverse method are compared in Figure 11. It can be seen that the reaction rate constants for the aforementioned three methods show the same trend with increasing the pyrolysis temperature; however, small differences in values of the reaction rate constants can be found. In order to carefully check these differences, the relative errors of NAMW of PEW at four measure points (N4, N5, N7, and N11) for the inverse method and screw-speed-dependent inverse method are shown in Table 4. Compared with the initial and modified Arrhenius equations in Table 3, both the inverse method and the screw-speed-dependent inverse method are of higher precision. It is meaningful that the monitoring precision is improved by 69.1% from the initial Arrhenius equation to the screw-speed-dependent inverse method.

## 5. Conclusions

Using a test platform, PEW was prepared from the pyrolysis of waste HDPE in a SSE and a connecting pipe. A program was developed in MATLAB to monitor NAMW of PEW from pyrolysis temperature and screw speed. The measured NAMWs of PEWs at measure points N4, N5, N7, and N11, which were not used as input data for the monitoring program, were compared with the calculated values to validate the monitoring. Our results showed that: (1) PEW of the controlled NAMW with a narrow melting peak was able to be prepared continuously from the pyrolysis of waste HDPE by extrusion; (2) the mathematical model by measure-point-independence verification showed good agreement with experimental results, and the average relative error of NAMW of PEW was not larger than 8.1% for the initial Arrhenius equation; (3) with the modified Arrhenius equation (*o* = 3), the average relative errors of NAMW of PEW for time-independent and time-dependent pyrolysis temperatures were similar and not larger than 3.59% and 3.58%, respectively; and (4) the average relative errors of NAMW of PEW for the inverse method and screw-speed-dependent inverse method were not larger than 3.1% and 2.5%, respectively, an indication that the monitoring program was able to precisely monitor NAMW of PEW from the pyrolysis of waste HDPE by extrusion.

## Figures and Tables

**Table 1:** The correspondence between pyrolysis time and screw speeds.

**Screw Speed (rpm)**	2	10	20	30	40
**Pyrolysis Time (s)**	842	213	179	146	110

**Table 2:** The arrangements of measure points under different values of *p*.

Pyrolysis Temperature (K)	673	693					713			733		
**Screw Speed (rpm)**	10	2	10	20	30	40	10	20	30	10	20	30
**NAMW of PEW**	4088	908	3111	3649	4268	5876	2919	3458	4151	2075	2445	3274
**Measure point**	N1	N2	N3	N4	N5	N6	N7	N8	N9	N10	N11	N12
***p* = 4**	✓	✓				✓				✓		
***p* = 6**	✓	✓				✓		✓	✓	✓		
***p* = 8**	✓	✓	✓			✓		✓	✓	✓		✓

**Table 3:** Relative errors of NAMW of PEW at four measure points (N4, N5, N7, and N11) for initial and modified Arrhenius equations.

Equation Order	Initial or Modified Arrhenius Equation	N4 (%)	N5 (%)	N7 (%)	N11 (%)	Mean (%)
*o* = 1	$\ln k(T_{p})=-3.6-4948.9/T_{p}$	5.8	10.4	8.0	8.3	8.1
*o* = 2	$\ln k(T_{p})=11.6-2.6\times 10^{4}/T_{p}+7.6\times 10^{6}/T_{p}{}^{2}$	6.6	11.3	7.1	7.2	8.0
*o* = 3	$\ln k(T_{p})=2.9\times 10^{3}-6.2\times 10^{6}/T_{p}+4.3\times 10^{9}/T_{p}{}^{2}-1.0\times 10^{12}/T_{p}{}^{3}$	2.2	6.7	0.8	4.7	3.6

**Table 4:** Relative errors of NAMW of PEW at four measure points (N4, N5, N7, and N11) for the modified Arrhenius equation (*o* = 3), inverse method, and screw-speed-dependent inverse method.

Relative Error (%)	N4	N5	N7	N11	Mean
modified Arrhenius equation (*o* = 3)	2.2	6.7	0.8	4.7	3.6
Inverse method	0.2	4.6	0.9	6.7	3.1
Screw-speed-dependent inverse method	0.6	4.8	0.2	4.5	2.5

## Nomenclature

*A*_1_:	the factor before exponent, s^−1^
*A*_2_:	the amplitude of temperature fluctuation, K
*b*:	the number of not pyrolyzed nodes
*d*:	search direction
*E*:	activation energy, kJ/mol
*J*:	objective function
*k*:	reaction rate constant
*l*:	the period of temperature fluctuation, s
*m*:	carbon-atom number
*M*:	the number-average molecular weight of polyethylene wax
$\bar{M}$:	the average carbon-atom number of moleculars of polyethylene wax
*n*:	screw speed
n:	the maximum carbon-atom number of moleculars of polyethylene wax
$N_{m}$:	the number of moleculars of polyethylene wax with *m* carbon atoms
N:	measure point (a test under a specific process condition)
*o*:	the equation order of initial or modified Arrhenius equation
*p*:	measure point number
*r*:	pyrolysis ratio
*R*:	gas constant (8.314 J/(mol·K))
*T*:	temperature, K
*t*:	time
*v*:	the order of reaction dynamic equation
*Z*:	the total number of input measure points
**Greek Symbols**	
β:	search step size
Δr:	the increment of pyrolysis ratio
ΔT:	temperature fluctuation, K
ε:	a small positive number
Ω:	computational domain
**Subscripts**	
cal:	calculated
m:	melting point
mea:	measured
p:	pyrolysis
v:	evaporation
**Superscripts**	
*i*, *j*:	iteration index
